# Circulating short chain fatty acids in Alzheimer's disease: A cross-sectional observational study

**DOI:** 10.1177/13872877251337773

**Published:** 2025-06-12

**Authors:** Moira Marizzoni, Luigi Coppola, Cristina Festari, Delia Luongo, Dominic Salamone, Daniele Naviglio, Andrea Soricelli, Peppino Mirabelli, Marco Salvatore, Annamaria Cattaneo, Giovanni B Frisoni

**Affiliations:** 1 Biological Psychiatry Unit, IRCCS Istituto Centro San Giovanni di Dio Fatebenefratelli, Brescia, Italy; 2 IRCCS SYNLAB SDN, Naples, Italy; 3 Laboratory of Neuroimaging and Alzheimer's Epidemiology, IRCCS Istituto Centro San Giovanni di Dio Fatebenefratelli, Brescia, Italy; 4 Istituto di Biostrutture e Bioimmagini (I.B.B.) - CNR, Naples, Italy; 5 Department of Agricultural Sciences, University of Naples Federico II, Portici, Italy; 6 Department of Chemical Sciences, University of Naples Federico II, Naples, Italy; 7 AORN Santobono-Pausilipon, UOS Laboratori di Ricerca e Biobanca, Naples, Italy; 8 Department of Pharmacological and Biomolecular Sciences, University of Milan, Milan, Italy; 9 Memory Clinic and LANVIE - Laboratory of Neuroimaging of Aging, University Hospitals and University of Geneva, Geneva, Switzerland

**Keywords:** Alzheimer's disease, cognitive impairment, microbiota-gut-brain axis, short chain fatty acids

## Abstract

Short chain fatty acids (SCFAs), produced mainly by gut microbes, might play a role in the pathophysiology of Alzheimer's disease (AD). We examined the SCFAs profile in 28 individuals with cognitive impairment due to AD (CI-AD), 29 with cognitive impairment not due to AD (CI-NAD), and 10 cognitively unimpaired (CU). CI-AD showed higher levels of acetate and valerate and lower levels of butyrate than CU and CI-NAD (p < 0.018). Acetate separated CI-AD from CI-NAD with AUC = 0.95 while the best neurodegeneration-related biomarker was GFAP with AUC = 0.79. SCFAs use for diagnosis and as treatment target in AD deserve further studies.

## Introduction

Gut bacteria might play a role in the pathophysiology of Alzheimer's disease (AD) through circulating metabolites. Short chain fatty acids (SCFAs), putative molecules mediating signals along the gut-brain axis, are mainly the result of anaerobic bacterial fermentation of indigestible carbohydrates or dietary fibers in the intestines.^1^ A small amount of SCFAs passes into the bloodstream and is transported to other body tissues where they mediate a wide range of biological functions, including host metabolism, immunity and appetite regulation.^1^

SCFAs have been implicated in the pathophysiology of AD in preclinical studies with inconsistent results. Some studies in AD mice reported a decrease in SCFA-producing bacteria compared with wild-type mice^2^ and the protective effect of the administration of butyrate^3^ or butyric acid-producing bacteria^4^ in reducing AD pathology. Another recent study in AD mice found that acetate facilitated neuroinflammation by inducing a proinflammatory microglia phenotype that ultimately led to increased hippocampal amyloid burden.^5^ Similarly, the administration of a cocktail of SCFAs to tauopathy model animals, induced hippocampal gliosis and tau pathology.^6^ We previously found that plasma SCFA levels (i.e., acetate, valerate and butyrate) were associated with brain AD pathology,^7^ but to the best of our knowledge, data comparing plasma SCFA level and biomarkers associated with neurodegeneration in AD patients and controls are lacking.

The aim of the present study was to assess the association of plasmatic SCFAs with a diagnosis of AD and neurodegeneration-related biomarkers.

## Methods

Participants were 85 community-dwelling individuals aged 50–85 recruited from a large Italian study on amyloid imaging in patients with cognitive complaints (Supplemental Method 1). In the context of this parent study, participants were proposed to contribute samples of stools and blood. Accepting patients signed an ad-hoc informed consent. Participants underwent the Mini-Mental State Examination (MMSE) and the Alzheimer's Disease Assessment Scale, cognitive portion (ADAS-Cog), and were not under antibiotic nor anti-inflammatory treatment over the past 3 months. [^18^F]-Florbetapir Amyloid PET was performed as previously reported.^7^ Amyloid positivity was defined as global standardized uptake value ratio versus cerebellum (SUVR) higher than 1.11. Plasma concentrations of the neurodegeneration-related biomarkers pTau-181 (V2 Advantage Kit; Cat. No. 103714), NFL (NF-Light immunoassay Advantage kit; Cat. N°103400) and of the neuroinflammatory-related marker GFAP^8^ (GFAP Human Discovery Kit; Cat. N° 102336) were measured using the ultrasensitive Simoa SR-X instrument (Quanterix, Lextington, USA). The levels of acetate, propionate, valerate and butyrate were tested using gas chromatography (GC) as previously reported.^7^

**Figure 1.**
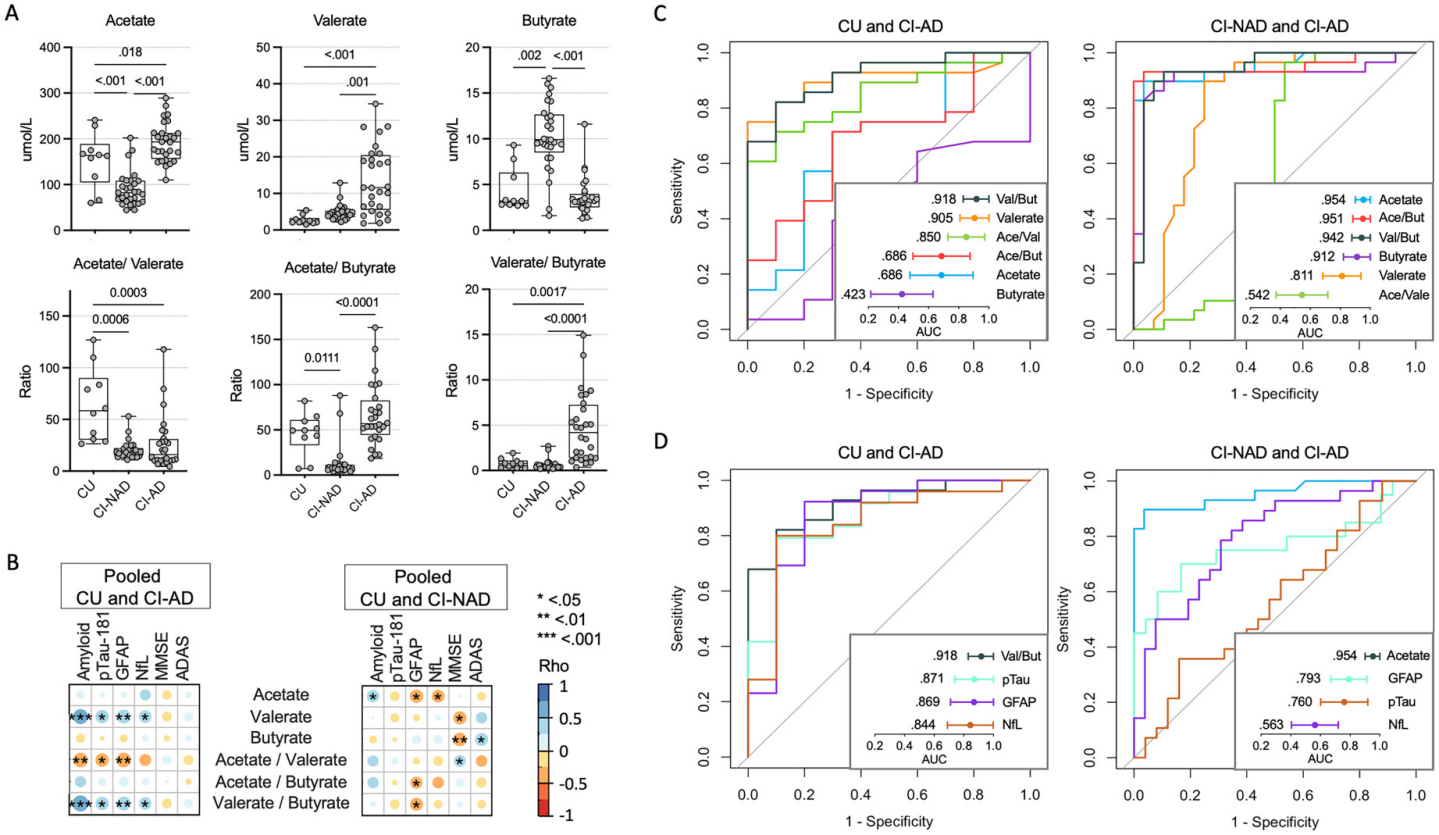
Plasma SCFA profile distinguished Alzheimer's individuals from CI-NAD and CU. Plasma SCFAs and their ratios (A). Association matrices between SCFAs and peripheral neurodegeneration-related biomarkers (B). Discriminative power of SCFAs and their ratios (C), and comparison between the best discriminative SCFA versus peripheral biomarkers with good evidence of validity for the AD diagnosis (D). CU: cognitively unimpaired persons; CI-NAD: patients with cognitive impairment not due to AD; CI-AD: patients with cognitive impairment due to AD.

Statistical analyses and figures were performed using RStudio (version 2023.03.1 + 446 with R version 4.3.0 (2023-04-21)), unless Figure 1A where GraphPad Prism (v10.2.3, GraphPad Software, San Diego, CA, USA) was used. Group comparison was performed using ANOVA with Bonferroni correction for continuous Gaussian variables, Kruskall-Wallis test with Dunn correction for non-Gaussian variables, Chi-square test for categorical data. Associations were assessed with Spearman correlation. Performance of SCFAs and peripheral neurodegeneration-related biomarkers in predicting diagnosis was calculated using receiver operating characteristic (ROC) curves (pROC package).^9^ DeLong's test included in the same R package was used to calculate the AUC 95% CI.

## Results

Demographic and clinical characteristics were as expected for this population, except for the high prevalence of hypertension within the CI-NAD (Table 1), suggesting that this group might be enriched by patients with vascular disease.

**Table 1.** Demographic and clinical features of study participants. Significant results (p < 0.05) are reported in bold.

	CU (N = 10)	CI-NAD (N = 29)	CI-AD (N = 28)	p	
				3-group ^b^	CI-NAD versus CI-AD ^c^
Age (y)	67.6 (6.6)	70.7 (7.0)	70.9 (6.2)	0.367	0.994
Female (N, %)	5 (50)	17 (59)	15 (54)	0.871	0.907
Education (y)	9.5 (5.5)	8.1 (3.7)	8.5 (4.4)	0.906	0.992
Body mass index^a^	24.1 (2.9)	25.6 (4.0)	24.6 (2.7)	0.364	0.499
*APOE* ε4 carrier status^d^ (N, %)	2 (25)	3 (10)	17 (61)	**<0**.**001**	**<0**.**001**
General cognition					
Mini-Mental State Examination	28.4 (1.3)	25.3 (3.3)	22.0 (5.5)	**<0**.**001**	**0**.**036**
ADAScog	8.24 (3.7)	14.3 (8.6)	17.2 (8.3)	**0**.**002**	0.117
Clinical stage (N, %)					
Mild cognitive impairment	-	19 (66%)	19 (68%)	NA	>0.999
Dementia	-	10 (34%)	9 (32%)	NA	0.819
Amyloid load (PET SUVr)	.95 (.07)	.94 (.07)	1.30 (.13)	**<0**.**001**	**<0**.**001**
Risk factors					
Hypertension	2 (20)	21 (72)	12 (43)	**0**.**007**	**0**.**047**
Diabetes	0 (0)	5 (17)	3 (11)	0.338	0.743
Vascular disease	2 (20)	10 (34)	2 (7)	**0**.**040**	**0**.**027**
Hypercholesterolemia	4 (40)	16 (55)	12 (43)	0.563	0.506
Number of cardiovascular risk factors	0 (0)	6 (21)	5 (18)	0.302	0.291
Drugs					
Acetylcholinesterase inhibitors	0 (0)	2 (7)	3 (11)	NA	0.967
Memantine	0 (0)	0 (0)	0 (0)	NA	NA
Antidepressant/hypnotic/anxiolytic	0 (0)	13 (45)	15 (54)	NA	0.693
Antipsychotic	0 (0)	1 (3)	1 (4)	NA	>0.999

^a^ weight/height^2^ and measured in kg/m^2^.

^b^ Statistical difference among the 3 groups by ANOVA for continuous Gaussian variables, Kruskal-Wallis for non-Gaussian variables, or Chi-squared test for categorical data.

^c^ p values of pairwise comparisons using Tukey correction for ANOVA and Benjamini-Hochberg correction for Kruskal-Wallis test.

^d^ Missing data for 2 CU participants.

CU: cognitively unimpaired persons; CI-NAD: patients with cognitive impairment not due to AD; CI-AD: patients with cognitive impairment due to AD; ADAScog: Alzheimer's Disease Assessment Scale, cognitive subscale; PET: positron emission tomography; SUVr: standardized uptake value ratio.

### Comparison of plasma SCFAs among groups and association with peripheral neurodegeneration- related biomarkers

SCFA profile analysis revealed two different signatures in CI-AD and CI-NAD (Figure 1A). CI-AD were characterized by elevated levels of acetate and valerate (p < 0.018 versus CU, p < 0.001 versus CI-NAD) while CI-NAD by reduced acetate and increased butyrate (p < 0.002 versus CU, p < 0.001 versus CI-AD). No significant results were reported for propionate. These changes were irrespective of *APOE* carrier status for all SCFAs except propionate (Supplemental Table 1), where the effect of clinical diagnosis emerged when adjusting the model for *APOE* carrier status and where CI-AD showed increased propionate levels compared with CI-NAD (p = 0.049, Supplemental Figure 1). With the acetate/valerate ratio, the difference between CU versus CI increased, regardless of the amyloid status (p < 0.0001), while those between the two groups of patients disappeared. The acetate/valerate ratio separated CI-NAD from CU (p < 0.011) and CI-AD (p < 0.0001) while the valerate/butyrate ratio separated CI-AD from CU (0.002) and CI-NAD (p < 0.0001).

Then, we tested the association of SCFAs with neurodegeneration-related biomarkers and global cognition (Figure 1B). In the correlation analyses including CU and CI-AD, valerate and its ratios were associated with amyloid and all the neurodegeneration-related biomarkers, more strongly with amyloid and plasma GFAP (0.45<|rho|<0.59; p < 0.007) and less with plasma pTau-181 and NfL (0.35<|rho|<0.42; p < 0.042). In the pooled CU and CI-NAD analyses, acetate was positively associated with amyloid (rho = 0.34, p = 0.035) and negatively associated with plasma GFAP and NfL (−0.40 < rho < -0.36; p < 0.026). In the analysis with CI-AD, no association between SCFA and cognitive measures was found. In contrast, the analysis including CI-NAD showed an association between high level of valerate and butyrate with low cognitive performance (0.29<|rho|<0.43; p < 0.048).

### Classification of CI-AD by plasma biomarkers

Next, we compared the area under the curves (AUC) of plasma SCFAs in identifying CI-AD versus CU and CI-NAD (Figure 1C). In the distinction between CI-AD and CU, valerate/butyrate ratio and valerate had very high discriminative power (AUC = 0.918, 95% CI: 0.830, 1000 and AUC = 0.905, 95% CI: 0.809, 1.000, respectively) as compared with the other SCFAs and ratios. In the classification of CI-AD versus CI-NAD, acetate and acetate/butyrate ratio showed the best discriminative power (AUC = 0.954, 95% CI: 0.901, 1.000 and AUC = 0.951, 95% CI: 0.885, 1.000, respectively) followed by valerate/butyrate ratio (AUC = 0.942, 95% CI: 0.877, 1.000) and butyrate (AUC = 0.912, 95% CI: 0.821, 1.00).

Lastly, we examined whether the performance of the best plasma SCFA was comparable to that of peripheral neurodegeneration-related biomarkers with good evidence of validity for the diagnosis of AD (Figure 1D). In the classification of CI-AD versus CU, the valerate/butyrate ratio had a slightly higher AUC compared to pTau-181 (AUC = 0.871, 95% CI: 0.738, 1.000). In the classification of CI-AD, acetate AUC was superior to GFAP (AUC = 0.793, 95% CI: 0.672, 0.913).

## Discussion

In this cross-sectional study on the evaluation of SCFAs in AD, we found that i) patients with cerebral amyloidosis showed a characteristic SCFA signature compared with those without amyloidosis and healthy controls and, ii) plasma valerate/butyrate ratio (AUCs of 0.918 versus CU) and acetate (AUC of 0.954 versus CI-NAD) classified CI-AD with very high accuracy, even better than that reported by plasma neurodegeneration-related biomarkers with good evidence of validity for the AD diagnosis.

To the best of our knowledge, we are the first to report analyses of plasma SCFAs in a human cohort and to show a peculiar SCFA profile in AD patients, specifically an increase in acetate and valerate compared with both cognitively healthy controls and patients with cognitive impairment not due to AD (Figure 1A). It should be noted that a weak association between clinical diagnosis and propionate levels emerged when the statistical model was corrected for *APOE* ε4 carrier status (i.e., CI-AD > CI-NAD), the main AD genetic risk factor known to impact the GM profile.^10,11^ Although *APOE* ε4 did not clearly affect the other SCFAs (Supplemental Table 1), future research investigating the impact of the *APOE* genotype on the association between SCFAs and brain pathology in the context of AD is necessary. The present findings are in conflict with the common thinking coming from AD preclinical studies and assigning SCFAs neuroprotective functions.^2–4^ However, our results are in agreement with two recent preclinical studies showing the association of SCFAs with AD pathology and neuroinflammation,^5,6^ and with a recent theory suggesting that the excess of these molecules can lead to AD,^12^ as in propionic acidemia, a metabolic disorder associated with brain atrophy, cognitive impairments, and dementia.^13^ Furthermore, increased saliva levels of acetate and propionate have been detected in AD patients^14,15^ and in persons with periodontal disease,^16^ a well-known risk factor for AD. Of note, a recent study applying ^11^C-acetate PET in AD, reported that ^11^C-acetate SUVR was elevated in MCI compared to CU and was associated with global and regional amyloid burden in MCI.^17^ Our data confirmed the hypothesis that each SCFA could have distinct effects and mechanisms of action in various contexts, such as in different diseases.^18^ Here, high levels of valerate were associated with AD pathology, neuroinflammation and neurodegeneration in CI-AD but not in CI-NAD. In CI-NAD, high levels of acetate were weakly associated with cerebral amyloid deposition as well as low neuroinflammation and neurodegeneration. Moreover, we found a direct association of valerate and butyrate with cognitive performance only in CI-NAD, suggesting that the link between SCFA unbalance and cognitive impairment in this group likely involved different pathways than AD. These results collectively suggest that the effect of SCFAs in mediating the association between GM dysbiosis and cognitive impairment might be mediated by amyloid and tau in CI-AD while it might act through more dynamic mediators (i.e., inflammation) in CI-NAD. For example, acetate supplementation appears to ameliorate inflammatory and neurodegenerative damage in preclinical models that do not develop amyloidosis or tauopathy, as in models of sleep disruption,^19^ Coffin-Siris syndrome^20^ and multiple sclerosis^21^ but can exacerbate the same phenomena in models that have amyloid and tau deposition.^5,6^ Although the causal link between SCFAs and cognitive impairment is far from being proven, these results strengthen the hypothesis that SCFAs have distinct effects and mechanisms of action depending on the disease.

Valerate/butyrate ratio and acetate have been identified as potential AD diagnostic biomarkers as they added value in distinguishing CI-AD cases from control subjects and CI-NAD as compared with plasma biomarkers proven to have good validity for the diagnosis of AD. Among AD blood-based biomarkers, the strong association of GFAP and pTau-181 with the diagnosis of AD replicated previous findings.^22,23^ Although promising, these results should be interpreted with caution since they were obtained from a small observational study and their replication in an independent, larger cohort is warranted. Another limitation of the study concerned the heterogeneity of the CI-NAD group. Since the original study design was intended to evaluate the diagnostic accuracy of amyloid PET in patients with cognitive complaints, the inclusion criterion referred only to the presence of cognitive impairment, regardless of diagnosis. Indeed, the CI-NAD group included patients with frontotemporal and cerebrovascular dementia as well as depression. Future studies on better characterized non-AD patient cohorts are needed to confirm the high diagnostic accuracy of SCFAs in differentiate CI-AD from CI-NAD.

To conclude, we report a novel association of SCFAs with AD pathology and neurodegeneration-related biomarkers suggesting that the gut microbiota might exert its action on the brain by modulating the levels of these circulating molecules. Acetate could improve the differential diagnosis of AD from other type of cognitive impairment and could improve the identification of amyloid status in patients who could benefit from anti-amyloid immunotherapies. Their use for diagnosis and as treatment target deserve further studies.

## Supplemental Material

sj-docx-1-alz-10.1177_13872877251337773 - Supplemental material for Circulating short chain fatty acids in Alzheimer's disease: A cross-sectional observational study Supplemental material, sj-docx-1-alz-10.1177_13872877251337773 for Circulating short chain fatty acids in Alzheimer's disease: A cross-sectional observational study by Moira Marizzoni, Luigi Coppola, Cristina Festari, Delia Luongo, Dominic Salamone, Daniele Naviglio, Andrea Soricelli, Peppino Mirabelli, Marco Salvatore, Annamaria Cattaneo and Giovanni B Frisoni in Journal of Alzheimer's Disease
