# Supplementary material for: Circulating short chain fatty acids in Alzheimer's disease: A cross-sectional observational study
Source: J Alzheimers Dis. 2025 Jun 12;106(1):38–43. doi: 10.1177/13872877251337773 (PMC12231880; doi:10.1177/13872877251337773)
Supplement: sj-docx-1-alz-10.1177_13872877251337773 - Supplemental material for Circulating short chain fatty acids in Alzheimer's disease: A cross-sectional observational study [file sj-docx-1-alz-10.1177_13872877251337773.docx]

**Supplemental Material**

**Circulating short chain fatty acids in Alzheimer’s disease: A cross-sectional observational study**

**Supplemental Method 1**

*Study participants*

Participants were community-dwelling persons of 50 to 85 years of age recruited from a large Italian study on amyloid imaging in patients with cognitive complaints, the Incremental Diagnostic Value of [^18^F]-Florbetapir Amyloid Imaging [INDIA-FBP] study.^1^ The inclusion criteria for participants with normal cognition were at most one neuropsychological test score outside the normal range. Cognitive impairment was defined as i) presence of cognitive complaints reported by patients or proxy or by the physician; ii) absence of intracranial metabolic or psychiatric causes of cognitive impairments; iii) presence of abnormal scores in ≥2 cognitive tests; and iv) history of progression of cognitive symptoms.

In the context of this parent study, 150 patients and controls who were not under antibiotic and anti-inflammatory treatment over the past 3 months or had been diagnosed with major depression or other psychiatric disorders, were proposed to contribute samples of stools and blood. Accepting patients signed an ad-hoc informed consent.

**Reference**

1. Boccardi M, Altomare D, Ferrari C, et al. Assessment of the incremental diagnostic value of Florbetapir F 18 imaging in patients with cognitive impairment: The incremental diagnostic value of amyloid PET with [18F]-Florbetapir (INDIA-FBP) study. *JAMA Neurol* 2016; 73: 1417–1424.

**Supplemental Table 1. *APOE* carrier status** **did not influence the SCFAs profile.** Two-way ANOVA comparison of the effect of *APOE* carrier status and clinical diagnosis on plasma SCFAs.

| **SCFA** | **Clinical Diagnosis** | ***APOE* carrier status** |
| --- | --- | --- |
| Acetate | F=40.67, ***p<0.001 | F=0.043, p=0.836 |
| Valerate | F=18.73, ***p<0.001 | F=2.81, p=0.100 |
| Butyrate | F=39.13, ***p<0.001 | F=1.90, p=0.173 |
| Propionate | F=0.038, *p=0.038 | F= 1.13, p=0.291 |

**Supplemental Figure 1.** Plasma propionate level of study participants stratified by diagnosis and analyzed using two-way ANOVA with Bonferroni correction including *APOE* carrier status and clinical diagnosis as factors.
